# Case report: Fatal cerebral herniation caused by hypoglycemic due to mistaking glibenclamide in children

**DOI:** 10.3389/fnbeh.2023.1085258

**Published:** 2023-03-23

**Authors:** Chunlan Song, Wanyu Jia, Shengli Shi, Peng Li

**Affiliations:** Children's Hospital Affiliated to Zhengzhou University, Henan Children's Hospital, Zhengzhou, China

**Keywords:** hypoglycemic coma, cerebral hernia, glibenclamide, mistake, children

## Abstract

In recent years, the prevalence of diabetes in China has significantly increased, and glibenclamide is widely used as a basic hypoglycemic drug in China's primary clinical practice. There are many left-behind children in the grass-roots areas in China and various dangerous incidents of children taking drugs by mistake continue to occur. This article reports a case of cerebral edema and fatal cerebral hernia caused by hypoglycemia induced by mistakenly ingesting glibenclamide in a child. This is the first reported case in China of a child who died from brain herniation caused by accidental administration of glibenclamide. This case reminded that clinicians must comprehensively consider the cause of convulsions and coma in children with unknown causes, ask the history in detail and cannot ignore the risk of hypoglycemic convulsions and coma. When hypoglycemic is detected, high concentration of glucose should be given promptly to normalize blood glucose. When dealing with unexplained convulsions and comatose children, clinical pediatricians must be alert to the possibility of accidental medication.

## 1. Introduction

The unintentional injury of children has become the leading cause of death in children. According to relevant data of the World Health Organization (WHO), more than 875,000 children and adolescents die of unintentional injury every year, among which poisoning is the fifth leading cause of death of children's unintentional injury (accounting for 5%; Hyder et al., 2009). According to the data released by Safe Kids Worldwide-China, drug poisoning is the leading cause of child poisoning (accounting for ~2/3 poisoning cases). Glibenclamide is widely used in China. This article reports of a case of hypoglycemia, coma, cerebral edema and fatal cerebral hernia caused by mistakenly ingesting glibenclamide in a child.

## 2. Case presentation

**Figure 1:**
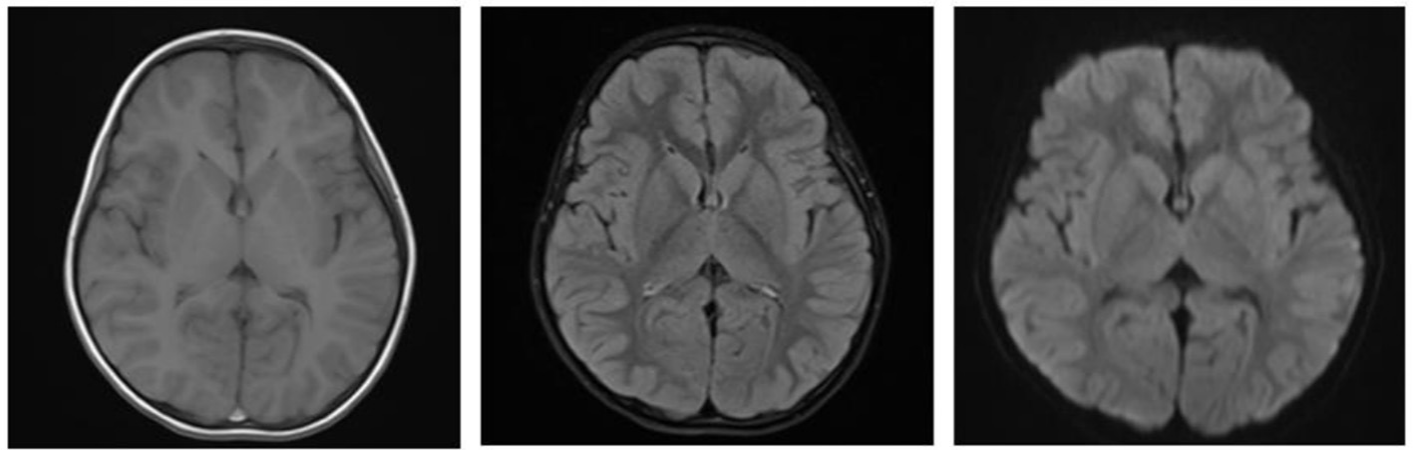
Cranial MRI on admission.

**Figure 2:**
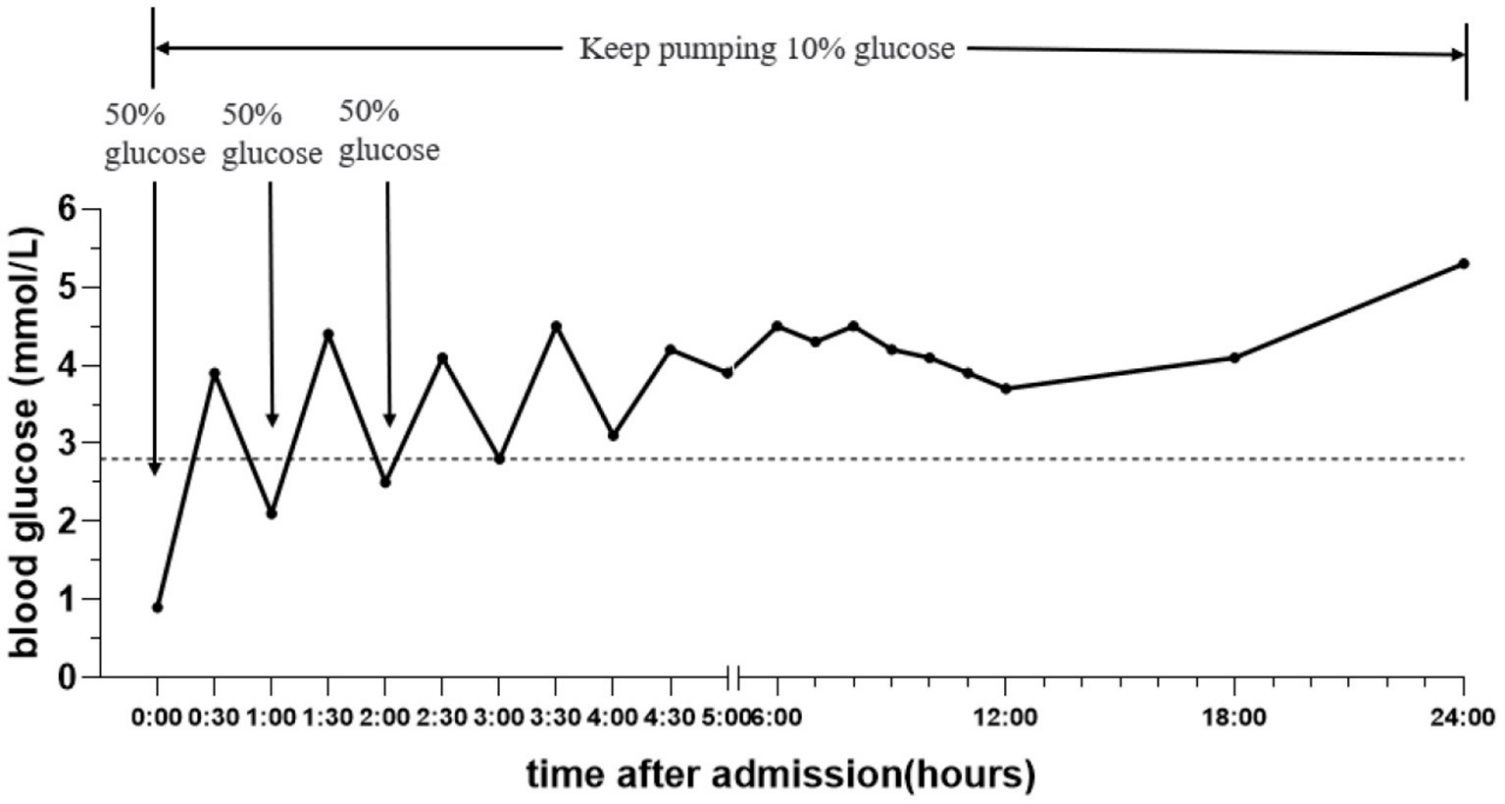
The changing trend of blood glucose concentration on the 1st day of admission.

A boy aged 3 years from Henan Province, China, was admitted to the Emergency Department of Henan Children's Hospital due to intermittent convulsions and unconsciousness for 15 h. The convulsions occurred three times of an unknown duration. In addition, the child experienced fecal incontinence and was comatose. The convulsions did not resolve after diazepam and chloral hydrate were administered to the child in the outpatient clinic, and the child was then admitted to the pediatric intensive care unit. The child's birth history, personal history, growth history and family history were not unique. Moreover, the physical examination revealed convulsions and superficial coma. The eyes were raised and staring, and the corners of the mouth were twitching. In addition, both upper limbs were shaking, and the hands were clenched. The bilateral pupils had a blunted light reflex. The perioral cyanosis was obvious, and the neck was tense with resistance. The muscle tone of the extremities was increased, muscle strength could not be accurately assessed and knee tendon reflexes were not elicited. The blood pressure was 105/56 mmHg. The blood glucose level was 0.9 mmol/L, and myocardial enzymes and markers of myocardial injury were above normal. S100 protein 0.161 ug/L and NES 80.55 ng/ml. Electrocardiogram and bedside chest X-ray results were not abnormal. Cerebrospinal fluid tests were essentially normal. The cranial MRI plain scan showed no abnormalities (Figure 1); the electroencephalogram suggested diffuse slow wave activity and paroxysmal epileptiform wave issuance during clinical symptom onset. Although the cranial MRI scan revealed no abnormalities for the time being, the child's frequent convulsions with impaired consciousness may cause cerebral ischemia and hypoxia, which can quickly lead to cerebral edema and increased intracranial pressure. This will further reduce cerebral blood flow even further and damages blood-brain barrier function, brain cell metabolism, and cerebrospinal fluid circulation, exacerbating cerebral edema and convulsions and creating a vicious circle. Thus, the child was immediately given a series of comprehensive treatments, including tracheal intubation, cardiac and blood pressure monitoring and intravenous medication to lower cranial pressure and to stop the convulsions after admission. Additionally, gastric lavage and diarrhea were given because poisoning couldn't be excluded. The child had difficulty correcting hypoglycemia and frequent convulsions. On the 1st day of admission, the child's hypoglycemia was difficult to correct and we gave several pushes of 50% glucose solution, along with a continuous infusion of 10% glucose solution and adjusted the pumping rate according to the child's blood glucose level (Figure 2). On the 2nd day after admission, the child's mother told the doctor about the possibility that the child was exposed to and mistakenly took glibenclamide. A blood specimen was immediately sent to a testing facility for glibenclamide testing, and the results were qualitatively positive. The diagnosis of glibenclamide poisoning was eventually confirmed.

**Figure 3:**
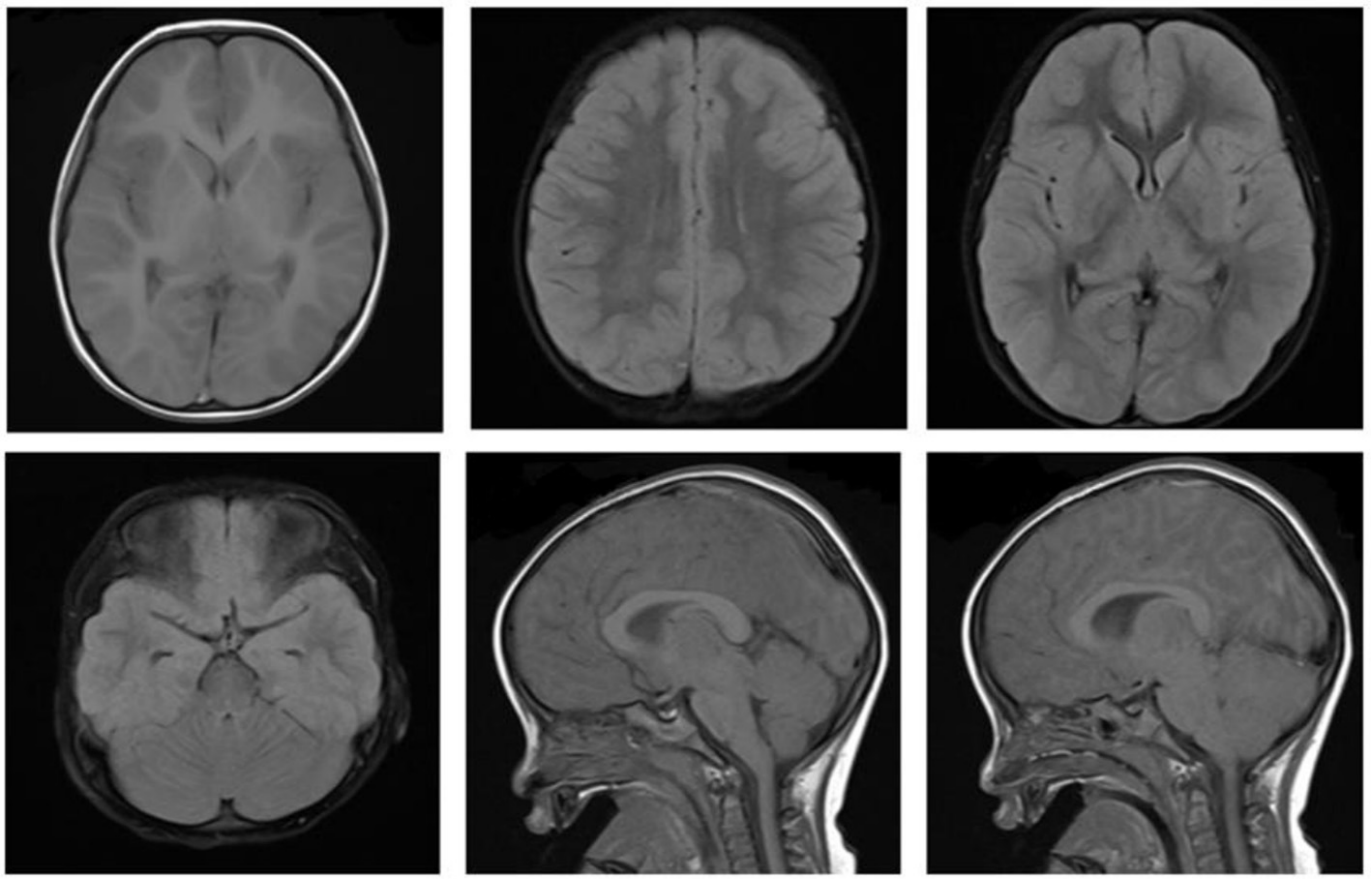
Cranial MRI on 4th day.

**Figure 4:**
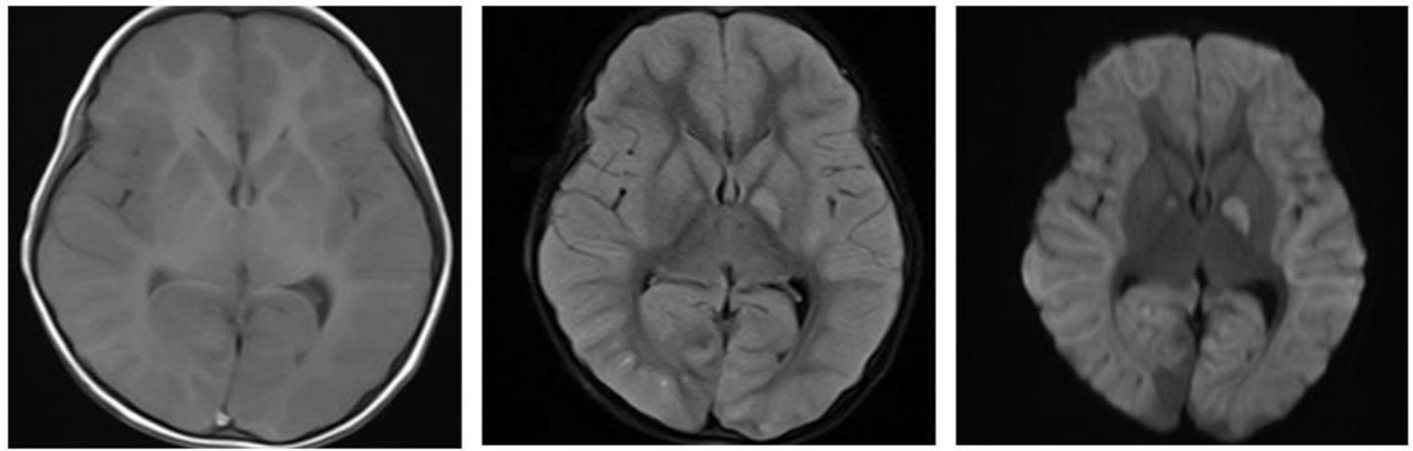
Cranial MRI on 10th day.

Immediately after the diagnosis, the child was given bedside hemoperfusion, during which time glucose was given, and blood glucose was dynamically monitored. However, his condition deteriorated, repeated convulsions. On the 3rd day of admission, the consciousness progressed into deep coma, bilateral pupils were unequal, the light reflex disappeared, Glasgow score was 3. On the 4th day of admission, cranial MRI was repeated: the bilateral cerebral hemispheres were swollen, with an extensive iso-T1 long T2 signal, a slightly high signal in FLAIR and a high signal in DWI in the bilateral cerebral hemispheres, hippocampus and basal ganglia. The bilateral temporal sulcus gyrus was deep into the suprasellar cisterna, the suprasellar cisterna was not clear and the brainstem was compressed. Bilateral ventricles were slightly narrowed, the suprasellar cisterna, prepontine cisterna and fourth ventricle were significantly narrowed and the position of the lower margin of the cerebellar hemisphere was moved down ~6.3 mm below the line of the foramen magnum, thus suggesting cerebral edema, cerebellar tonsillar hernia, and bilateral temporal sulcus hernia (Figure 3). The cranial MRI was repeated at 10 days after admission, the bilateral cerebral hemispheres were still swollen, the images were essentially the same as the previous analysis, suggesting increased cerebral edema, cerebellar tonsillar hernia and bilateral temporal sulcus hernia (hernias were worse than the anterior one; Figure 4). The blood pressure fluctuated from 83–147/45–71 mmHg during the course of treatment, which was unstable, possibly due to cerebral edema, brain herniation. The child was critically ill and poorly treated; the parents abandoned treatment after 3 weeks of treatment, and the child died after the tracheal tube was removed, and the ventilator was evacuated. The death diagnosis: 1. Cerebral hernia (foramen magnum hernia, temporal sulcus hernia); 2. Status epilepticus; 3. Drug poisoning (glibenclamide); 4. Hypoglycemia, brain damage; 5. Multiple organ function injury.

## 3. Discussion

The prevalence of diabetes in China has significantly increased, and type 2 diabetes accounts for more than 90% of cases (Society, 2021). Sulfonylureas is one of the most widely used oral hypoglycemic agents (Colagiuri et al., 2018; Douros et al., 2018). Glibenclamide, a second-generation oral hypoglycemic agent of sulfonylureas (also known as euglycemia), is widely used in the treatment of diabetes because of its low price and obvious clinical efficacy. The main pharmacological effect of glibenclamide is to stimulate insulin secretion from pancreatic beta cells of the pancreas to induce lowering of blood glucose (Baofeng et al., 2018). The most obvious side effect of glibenclamide is hypoglycemia (Gangji et al., 2007; Douros et al., 2018). Glibenclamide is rapidly absorbed orally, with a protein binding rate up to 99%, and the blood concentration peaks 2–6 h after oral administration and continues to act for 24 h. The half-life is about 10 h (Pearson, 1985). Glibenclamide has a relatively long terminal half-life in chronic dosing compared with other sulfonylureas, owing to its high affinity for the β-cell sulfonylurea receptor and the accumulation of active metabolites that are excreted *via* the kidney (Gangji et al., 2007). Some patients continue to experience hypoglycemia even after stopping glibenclamide; in severe situations, this can cause comas.

The clinical manifestations of hypoglycemia are manifested in two main areas: autonomic and brain dysfunction (Nakhleh and Shehadeh, 2021). When blood glucose levels fall, the secretion of glucose-raising hormones (such as adrenaline and glucocorticoids) will increase. This causes sympathetic excitement in the body, which manifests as autonomic symptoms including anxiety, tremors, palpitations, diaphoresis, paresthesia and sensation of hunger. Glucose provides nearly all of the energy required by the brain tissue. Once hypoglycemia persists, electrolyte imbalance in the brain tissue, neurotransmitter metabolism disorders and blood-brain barrier destruction result in clinical symptoms of brain dysfunction, such as headache, dizziness, slow reaction, blurred vision, backward thinking, unclear language, drowsiness, unsteady walking, loss of vision, impaired consciousness, limb paralysis, tremors, restlessness, and loss of consciousness (Nakhleh and Shehadeh, 2021). Additionally, some hypoglycemic patients will begin to convulse and present with symptoms that resemble focal epilepsy, which could lead to a false positive diagnosis. The differential diagnosis relies on an in-depth analysis of the EEG or a video EEG (Dudley et al., 2022). Hypoglycemic coma or even irreversible brain damage can occur as the disease progresses. Hypoglycemia-induced severe brain dysfunction can result in life-threatening cerebral infarction, cerebral edema, a rapid increase in intracranial pressure, brain herniation and brain death. Consequently, when glibenclamide is clinically used to treat diabetes mellitus, it must be rationally administered while dynamically monitoring blood glucose, with special attention given to the prevention and vigilance of hypoglycemia and hypoglycemic coma.

For patients in a hypoglycemic coma, timely analysis to identify the cause and treatment for correcting hypoglycemia is crucial. Glibenclamide-induced hypoglycemia has mostly been reported in adults with diabetes (Leblanc et al., 2004; Hussain et al., 2016; Douros et al., 2018). The treatment of severe hypoglycemia is divided into three major aspects. (1) The correction of hypoglycemia. Supplemental glucose can be provided (Nakhleh and Shehadeh, 2021), which can be given orally to conscious patients and intravenously to patients with consciousness impairments. Intravenous glucose was initially given at a concentration of 50%, and the patient was given the glucose intravenously at a concentration of 10% after waking up. The condition was closely observed for at least 1 day. Glucocorticoids and glucagon can be considered when hypoglycemia is difficult to correct. (2) The removal of residual drugs. For example, gastric lavage, drainage, blood perfusion, and others. (3) Intravenous mannitol can be considered for reducing intracranial pressure while protecting organ function and maintaining the stability of the internal environment in patients with persistent episodes of hypoglycemic coma. In children with acute intracranial pressure elevation, timely identification, attention to comprehensive management, and reasonable selection of cranial pressure-lowering measures to ensure normal cerebral perfusion are of great clinical significance. Surgery may be an option for children whose cranial pressure has not significantly decreased after receiving conventional comprehensive cranial pressure lowering therapy, but more research is still needed to determine the medium- and long-term effects of decompressive craniectomy on children with cranial hypertension (Luo and Li, 2019).

The oral administration of glibenclamide in children leads to hypoglycemic coma mostly due to misuse of the drug. Currently, in China, diabetes in children and adolescents is still predominantly in the form of T1DM, accounting for ~85–90% of childhood diabetes mellitus (Society, 2021). The treatment of children with T1DM has mainly involved a combination of exercise, nutrition and subcutaneous insulin injections. Oral glibenclamide treatment has rarely been used in the treatment of diabetes in children. Sulfonylurea ingestion is life-threatening in toddlers due to its strong and prolonged hypoglycemic effects (Glatstein et al., 2009). Tian Yunfan et al. reported of a case of blindness and neurological sequelae in a young child who was aggressively treated for glibenclamide-induced hypoglycemic coma (Yunfen and Li, 2008).

In this case, we reported a case of coma and brain herniation caused by the misuse of glibenclamide, which eventually led to death. The tragic lessons learned will hopefully draw attention to the situation. We summarized some enlightenment and lessons brought by the case, which we hope can draw the attention of medical practitioners. (1) The child was a preschooler, a non-diabetic patient, and started with convulsions without obvious other accompanying symptoms. Therefore, the first doctor's treatment focused on relieving the convulsions and did not fully consider the possibility of neurological symptoms caused by hypoglycemia, resulting in the child's intermittent convulsions and coma for 15 h when he was transferred to our hospital. The duration of hypoglycemic coma was too long. Some studies have shown that brain tissue damage is irreversible after more than 6 h of continuous hypoglycemic episodes. This suggested that we should consider the convulsions caused by hypoglycemic encephalopathy as one of the differential diagnoses when receiving children with convulsions or coma, and monitor the blood glucose level in a timely manner. (2) The child presented with persistent hypoglycemia after admission. Although we gave high concentration of glucose in time to restore his blood glucose level to normal, we did not know enough about the etiology of persistent hypoglycemia. The cause of persistent hypoglycemia was not clearly identified after spending a lot of time in the early stage. The diagnosis of glibenclamide misuse was confirmed only after detailed medical history and blood test, and bedside blood perfusion was performed. This suggests that we need to consider the misuse of glucose-lowering drugs when we encounter a non-diabetic patient with persistent hypoglycemia and pursue the relevant information in detail, and if necessary, perform blood drug concentration tests to assist in the diagnosis. (3) In this case, the child took the medication by mistake while playing, which eventually led to the tragedy, and the parents failed to provide relevant medical history in time during the treatment. Children are at high risk of drug misuse, so pediatricians should repeatedly ask for medical history when they see children suspected of drug misuse to identify the cause early and formulate a scientific and reasonable treatment plan at an early stage to avoid serious harm to the children. The best treatment for similar diseases is prevention, and we need to strengthen scientific education and awareness for child caregivers. For families with children who require long-term oral administration of drugs such as glibenclamide, it is important to keep the medications properly and try to avoid drug abuse. (4) Hypoglycemia is a symptom that is easily overlooked in daily life and is generally under-recognized by the population, especially in children whose hypoglycemia symptoms are less typical compared to adults, but persistent hypoglycemia can still cause serious consequences. We suggest medical units to strengthen the education of the public and medical personnel to popularize the symptoms, hazards and treatment methods related to hypoglycemia.

## Data availability statement

The original contributions presented in the study are included in the article/supplementary material, further inquiries can be directed to the corresponding author.

## Ethics statement

Written informed consent was obtained from the individual(s), and minor(s)' legal guardian/next of kin, for the publication of any potentially identifiable images or data included in this article.

## Author contributions

PL contributed to conception and design of the study. CS and WJ wrote the first draft of the manuscript. SS contributed to the acquisition and analysis of data for the work. All authors contributed to manuscript revision, read, and approved the submitted version.
